# When Fear Meets Joy: Cultural Differences in the Impact of Decision Uncertainty on Fear of Better Options and Ditto Consumption

**DOI:** 10.3390/bs16060849

**Published:** 2026-05-26

**Authors:** Haoyue Bai, Junghee Kim, Seolwoo Park

**Affiliations:** 1 College of Business Administration, Lanzhou University of Finance and Economics, Lanzhou 730101, China; 2 Department of Business Administration, Jeju National University, Jeju 63243, Republic of Korea

**Keywords:** decision uncertainty, fear of better options, ditto consumption, joy of missing out, fear of missing out, cultural differences

## Abstract

This study examines how decision uncertainty shapes consumers’ Fear of Better Options (FOBO), and subsequently is associated with ditto consumption, while assessing FOBO’s mediating role and the moderating effects of emotional state (Fear of Missing Out, FOMO/Joy of Missing Out, JOMO) and cultural differences (China/Korea). Using survey data from 682 new energy vehicle consumers in China and Korea, structural equation modeling was applied to test the proposed framework. The results reveal that choice overload and price fluctuation significantly increase both FOBO and ditto consumption, while obsolescence risk does not show a significant direct effect. Notably, time pressure negatively influences FOBO but positively affects ditto consumption, suggesting a dual-path mechanism in decision-making under time constraints. FOBO partially mediates the effects of choice overload and price fluctuation on ditto consumption. Moreover, emotional state and cultural differences moderate these relationships: FOMO amplifies, whereas JOMO mitigates the transmission effect of FOBO. Chinese consumers display stronger overall effects compared with their Korean counterparts. This study expands upon uncertainty avoidance theory by incorporating FOBO into consumer decision-making models, providing insights into how decision uncertainty, along with cultural and emotional factors, can inform marketing strategies.

## 1. Introduction

In the rapidly evolving commercial landscape of the digital economy era, the accelerating product iteration pace, exponentially shortened price fluctuation cycles, and progressively narrowing consumer decision windows collectively generate a multidimensional stress matrix, thereby shaping a high-load consumption scenario characterized by choice overload (Adriatico et al., 2022; Hassen, 2024; Wu et al., 2025). This dynamic environment has reshaped consumer decision psychology, where the perception of “Never Rush, Always Save” increasingly prevails (Rautela & Sharma, 2022; Tan et al., 2025; Trupia & Shaddy, 2025). Facing non-linear transformations in supply-demand relationships, consumers have developed an emerging psychological phenomenon termed “Fear of Better Options (FOBO)” during decision-making processes. FOBO refers to individuals’ tendency to delay or abandon purchases due to perceived availability of superior alternatives, which not only prolongs decision-making duration and increases cognitive load but may also lead to decision paralysis (Flecha Ortiz et al., 2024; Park & Kim, 2025). Closely related to FOBO is the behavioral tendency known as ditto consumption, where consumers rely on others’ choices to reduce uncertainty when facing decision dilemmas (Kim, 2023). This ditto-driven consumption pattern has become increasingly pronounced through the amplification of social media, influencer marketing, and online review systems. However, does this imitation truly alleviate FOBO? Or is it merely another form of decision procrastination that ultimately deepens consumer anxiety?

Recent market observations in the new energy vehicle sector further illustrate how decision uncertainty manifests in high-involvement consumption. Consumers navigating the rapidly expanding arrays of models and brands in China and South Korea are often overwhelmed by competing claims on battery range, autonomous driving features, and volatile government subsidies, uncertain about whether their final choice is truly the most future-proof and cost-effective option available (Escobar-Miranda & Ballesteros-Sánchez, 2025; Xue et al., 2025). This sense of hesitation frequently relates to FOBO, a persistent worry that a technologically superior or better-valued vehicle is being overlooked (Park & Kim, 2025; Tan et al., 2025). Meanwhile, some individuals cope through ditto consumption, relying on sales rankings, mainstream brand reputations, or the recommendations of key opinion leaders to ease the anxiety of making a regrettable investment (Flecha Ortiz et al., 2024). Thus, while FOBO reflects a consumer’s hesitation to commit, driven by the concern that superior options exist, ditto consumption represents a compensatory strategy to minimize cognitive burden by following the majority’s choices. These behaviors demonstrate that decision uncertainty is not merely an abstract concept but a critical factor influencing high-stakes purchases in key Asian markets.

However, in contrast to the vivid market phenomena and sophisticated coping strategies observed in practice, academic research in explaining the underlying cognitive mechanisms and boundary conditions still exhibits three critical gaps. First, existing theoretical perspectives remain fragmented. Although prior studies have shown that decision uncertainty intensifies consumer anxiety, induces choice deferral (Flecha Ortiz et al., 2024; Yang et al., 2021), and may lead to decision paralysis under choice overload (Adriatico et al., 2022; Gaur et al., 2023; Hassen, 2024), this literature has predominantly focused on the basic uncertainty to deferral pathway. How FOBO functions as a central psychological mechanism that translates decision uncertainty into downstream behavioral tendencies has not yet been systematically theorized or empirically validated. Second, research paradigms often operate in isolated silos. While external market volatilities are known to amplify decision pressure (Alnes & Haugom, 2024; Begho & Liu, 2024), and internal cognitive loads can increase decision difficulty (Bobadilla-Suarez & Love, 2018; Liu et al., 2022; Wu et al., 2025), the majority of investigations examine these factors’ effects in isolation. Consequently, it remains unclear whether these factors interact through FOBO to amplify anxiety or, alternatively, trigger avoidance-oriented decision paths. Third, the cross-cultural perspective adopted in prior work lacks granularity and contextualization. Macro-level Hofstede-style comparisons often overlook subtle yet meaningful distinctions within East Asian cultures, particularly differences between China and Korea in social trust structures and uncertainty-avoidance tendencies (Bai & Kim, 2024; Deng & Gao, 2025; Kang et al., 2020; Lee et al., 2022; Truong, 2025). Moreover, emotional states such as FOMO and JOMO (Eitan & Gazit, 2024; Park & Kim, 2025; Tan et al., 2025), which may serve as important contextual boundary conditions, have received insufficient attention within existing cross-cultural frameworks.

Addressing these gaps, this study pursues four objectives. First, it analyzes how decision uncertainty factors (obsolescence risk, choice overload, price fluctuation, and time pressure) influence FOBO and ditto consumption. Second, it explores FOBO’s mediating role in consumer decision-making, particularly how it is associated with consumers’ reliance on others’ choices, which in turn is associated with ditto consumption behavior. Third, it examines the moderating effects of emotional state (FOMO/JOMO) and cultural differences (China/Korea) on the proposed mechanisms, revealing variations in consumer coping strategies and behavioral differences across cultural contexts. Fourth, it constructs a comprehensive theoretical framework to provide new insights into consumer decision uncertainty and imitation behavior for academic research while offering theoretical and practical guidance for businesses and marketers on optimizing consumer experience and alleviating decision anxiety. By enriching the theoretical framework of consumer decision-making, this study also provides empirical support for businesses in product marketing, pricing strategies, and consumer experience optimization.

## 2. Literature Review

### 2.1. Decision Uncertainty

Decision uncertainty refers to consumers’ perceived difficulty in making choices when facing incomplete information, unpredictable outcomes, or cognitive limitations that hinder confident evaluation (Adriatico et al., 2022; Guillard et al., 2023). According to Uncertainty Avoidance Theory, heightened uncertainty increases risk aversion, prompting hesitation, deferral, or reliance on simplified choice strategies (Yang et al., 2021). Prior studies indicate that decision uncertainty can arise from multiple sources within the choice environment, including obsolescence risk, choice overload, price fluctuation, and time pressure (Acikgoz et al., 2025; Alnes & Haugom, 2024; Chernev et al., 2015; Wu et al., 2025). These conditions disrupt consumers’ ability to form stable preferences and intensify their fear of making suboptimal decisions, thereby shaping FOBO and downstream behavioral responses. Although these four sources differ in nature, they collectively converge on a common psychological challenge, undermining consumers’ confidence in making a satisfactory choice, and thus can be understood as contextual manifestations of decision uncertainty within a unified theoretical framework (Yang et al., 2021).

Obsolescence risk refers to the consumer’s perception that a product may soon become outdated or devalued due to rapid technological innovation or the launch of superior models (Acikgoz et al., 2025). In the context of consumer behavior, it triggers a protective, anxiety-driven response, as consumers weigh current options against anticipated future improvements (Becher & Sibony, 2021). This form of uncertainty directly intensifies FOBO by reinforcing the belief that a “better option” is always on the horizon, thereby prolonging decision delays and heightening the fear of making a suboptimal choice (Kang et al., 2020; Demirel, 2025). Moreover, consumers facing high obsolescence risk increasingly rely on social validation to mitigate perceived decision uncertainty, leading them to engage in ditto consumption behaviors such as following popular trends, choosing best-selling products, or adopting peer recommendations to reduce complexity and emotional burden (Guillard et al., 2023).

Choice overload refers to consumers’ decision difficulty, delay, or abandonment caused by excessive options and cognitive overload (Adriatico et al., 2022; Jacob et al., 2024). Rooted in Cognitive Load Theory and the Paradox of Choice, it suggests that too many alternatives lower satisfaction and provoke decision paralysis or regret (Hassen, 2024). Choice overload reinforces FOBO by creating the illusion of better alternatives, hindering final decisions. Scheibehenne et al. (2010) found that excessive options often lead consumers to postpone decisions or revert to habitual choices. Such hesitation is especially prominent in the electric vehicle market, where abundant choices and price comparisons amplify FOBO (Jacob et al., 2024). To reduce cognitive burden under choice overload, consumers often conform by following the majority, selecting best-sellers, or relying on opinion leaders (F. Wang et al., 2021).

Price fluctuation refers to the instability of product or service prices over time, driven by supply-demand dynamics, competition, and dynamic pricing strategies (Jeong et al., 2019). In consumer behavior research, price fluctuation represents external uncertainty that affects perceived value, purchase decisions, and price fairness perceptions (Alnes & Haugom, 2024). Frequent price changes trigger loss aversion, encouraging consumers to delay purchases or wait for discounts (Bai & Kim, 2024). Dynamic pricing suggests that frequent price fluctuations create psychological dilemmas, prompting consumers to postpone purchases in hopes of securing better deals (Alnes & Haugom, 2024; Fennell et al., 2025). Additionally, prospect theory suggests that consumers perceive losses from overpaying more strongly than gains, reinforcing decision delay tendencies (Kahneman & Tversky, 2013). Gaur et al. (2023) found that in uncertain pricing environments, consumers often use social validation as a coping mechanism, relying on peer recommendations and product ratings to guide their decisions.

Time pressure refers to the urgency individuals experience when making decisions under externally imposed time constraints (Wu et al., 2025). It shapes information processing by requiring decisions within a limited time, often leading to rapid but incomplete decisions, reliance on heuristics, and increased risk of errors (Bobadilla-Suarez & Love, 2018; Liu et al., 2022). Consumers under time pressure often reduce information search and rely on social proof or ditto consumption to simplify decisions (Begho & Liu, 2024; Wu et al., 2025). Bai and Kim (2024) further suggest that time-limited discounts significantly influence purchase decisions. Accordingly, time pressure may lower decision quality, increase regret, and affect satisfaction and purchasing behavior.

In summary, despite their distinct operationalizations, obsolescence risk, choice overload, price fluctuation, and time pressure all contribute to heightened decision uncertainty, which in turn triggers FOBO and ditto consumption through shared psychological mechanisms. Based on the above discussion, this study proposes the following hypotheses:

**H1.**  *Decision uncertainty (a. obsolescence risk, b. choice overload, c. price fluctuation, d. time pressure) has a significant positive effect on FOBO.*

**H2.**  *Decision uncertainty (a. obsolescence risk, b. choice overload, c. price fluctuation, d. time pressure) has a significant positive effect on ditto consumption.*

### 2.2. Fear of Better Options

Fear of Better Options (FOBO) refers to consumers’ hesitation in decision-making driven by the belief that better options may exist (Park & Kim, 2025). Unlike the socially oriented Fear of Missing Out (FOMO), which centers on anxiety about missing others’ rewarding experiences (Eitan & Gazit, 2024), FOBO is rooted in the contemplation of product alternatives regardless of peer influence. Unlike traditional decision-making biases, FOBO reflects both risk aversion and consumers’ desire to maximize utility amid unlimited choices (Hassen, 2024). It induces stress and post-purchase regret, yet unlike the future-oriented anticipated regret (Kahneman & Tversky, 2013), FOBO captures present-moment hesitation over whether a superior option might be just around the corner. This is associated with decision avoidance and delay, but FOBO is distinguished from mere behavioral delay as it represents the underlying psychological state of indecision rather than just the act of postponing a choice (Park & Kim, 2025). FOBO’s specific focus is on the perceived existence of a superior alternative in the immediate choice context. This present-oriented belief reduces confidence in one’s own judgment and increases the appeal of following others’ validated choices (Kang et al., 2020; Flecha Ortiz et al., 2024). In contrast, anticipated regret (He et al., 2026) is future-oriented and does not inherently motivate immediate imitation; maximization tendency (Ma et al., 2023) is a stable disposition that does not vary situationally; and decision delay is merely a behavioral outcome and does not explain why consumers turn to social cues rather than simply postponing (Park & Kim, 2025). High FOBO levels prompt consumers to avoid responsibility and seek validation through collective choices (Flecha Ortiz et al., 2024). This shift reflects a coping mechanism, reducing anxiety through social conformity in uncertain environments. As a psychological barrier, FOBO amplifies dependence on ditto consumption, driving consumers to follow peer choices to relieve anxiety (Kang et al., 2020). Under uncertainty, individuals may postpone purchases or adopt ditto consumption to reduce cognitive load. In other words, consumers are more inclined to trust collective wisdom and simplify decision-making by imitating others’ choices. Based on this, the following hypotheses are proposed:

**H3.**  *FOBO has a significant positive effect on ditto consumption.*

**H4.**  *FOBO mediates the relationship between decision uncertainty (a. obsolescence risk, b. choice overload, c. price fluctuation, d. time pressure) and ditto consumption.*

### 2.3. Ditto Consumption

Ditto consumption refers to consumer behavior in which individuals cope with decision overload by imitating others’ choices (Kahneman & Tversky, 2013). The term “Ditto”, derived from the Latin word meaning “same as above” or “following the crowd”, conceptualizes this herd consumption behavior, which was officially recognized as a top consumer trend by the 2024 Korea Consumer Trend White Paper (Kim, 2023). While this behavior resonates with long-established concepts such as the bandwagon effect and reference group influence, it is critical to delineate its conceptual boundaries in the modern context. Traditional theories of herd behavior often emphasize a passive conformity driven by normative social pressure, the desire to fit in and avoid social sanctions (Begho & Liu, 2024). The bandwagon effect similarly captures a tendency to adopt what is popular, but without a specific focus on uncertainty reduction (Mai et al., 2026). In contrast, ditto consumption, particularly in the context of modern, high-involvement purchases, manifests as a more active, information-driven, and utilitarian heuristic strategy. When faced with overwhelming choice overload, rapid price fluctuations, or the FOBO, consumers are not merely passively succumbing to social norms. Instead, they are proactively employing the choices of others as a decision-making shortcut to reduce cognitive effort, mitigate perceived risk, and efficiently resolve the anxiety induced by decision uncertainty (Kang et al., 2020; J. Wang et al., 2023). Thus, unlike passive conformity or simple popularity-seeking, ditto consumption is specifically triggered by decision uncertainty and serves a clear anxiety-reduction function. According to social identity theory, individuals use others’ choices as a reference when facing decision uncertainty, leading to imitation-based decisions (Begho & Liu, 2024). In e-commerce, hesitant consumers rely on reviews and rankings to reduce anxiety (Flecha Ortiz et al., 2024). Additionally, Begho and Liu’s (2024) study has indicated that consumers exhibit a high tendency toward herd behavior when purchasing food. As a heuristic shortcut, ditto consumption allows quick decisions while minimizing cognitive effort (J. Wang et al., 2023).

### 2.4. Emotional State

Joy of Missing Out (JOMO) and Fear of Missing Out (FOMO) represent two opposing emotional states in consumer behavior (Chan et al., 2022; Wibowo & Safaria, 2025). Specifically, JOMO reflects satisfaction with personal choices and deliberate disengagement from external influence (Barry et al., 2023), whereas FOMO manifests as anxiety over missed opportunities and an obsessive focus on social or market trends (Eitan & Gazit, 2024). Conceptually, these two states are antithetical; an individual cannot simultaneously experience high levels of both JOMO and FOMO. This inherent opposition makes them suitable for treatment as a single bipolar continuum, where the relative dominance of one over the other determines the net emotional orientation (Eitan & Gazit, 2024; Li & Han, 2025; Rautela & Sharma, 2022). Existing research suggests that JOMO alleviates FOBO by enhancing confidence in existing decisions, while FOMO exacerbates FOBO by increasing sensitivity to potential losses and alternatives (Al-Ansi et al., 2024; Kang et al., 2020). Within ditto consumption, JOMO weakens the link between FOBO and ditto consumption by reducing choice anxiety, as consumers with high JOMO derive satisfaction from delaying decisions and are less driven by social validation (Rautela & Sharma, 2022). In contrast, FOMO strengthens this relationship, as consumers with high FOMO experience greater pressure to conform and tend to imitate popular choices to avoid regret (Tan et al., 2025; J. Wang et al., 2023). Therefore, JOMO and FOMO exert opposite moderating effects on the relationship between FOBO and ditto consumption. Accordingly, the following hypotheses are proposed:

**H5.**  *FOMO strengthens the positive effect of decision uncertainty (a. obsolescence risk, b. choice overload, c. price fluctuation, d. time pressure) on FOBO, whereas JOMO weakens this effect.*

**H6.**  *In the mediating pathway of decision uncertainty (a. obsolescence risk, b. choice overload, c. price fluctuation, d. time pressure) affecting ditto consumption via FOBO, emotional state further moderates the relationship between FOBO and ditto consumption.*

### 2.5. Cultural Differences

Cultural factors critically shape consumer decision-making, especially regarding uncertainty avoidance, social conformity, and decision-making strategies (Al Mubarak & Giouvris, 2024; Bai et al., 2024). Although both China and South Korea are collectivist societies, a nuanced examination reveals consequential differences in their cultural profiles (Bai & Kim, 2024; Shin, 2025). An analysis of Hofstede Insights data substantiates this view, revealing critical divergences in dimensions highly relevant to consumption under uncertainty. Most notably, China exhibits a significantly higher Long-Term Orientation score (87) compared with South Korea (75), indicating a stronger pragmatic focus on future-proofing. Furthermore, South Korea’s classification as a Restraint society (29) compared with China’s more Pragmatic–Indulgent stance (42) suggests a greater skepticism towards gratification and a stronger reliance on social norms for risk mitigation. These macro-level dimensions can be further refined using the vertical/horizontal collectivism framework (Sharon et al., 2008), which distinguishes between status-oriented, competitive collectivism (vertical) and harmony-oriented, in-group focused collectivism (horizontal). These macro-level dimensions are reflected in distinct micro-level consumer behaviors. In China, which leans toward vertical collectivism, the widespread penetration of social commerce, key opinion leaders, and key opinion consumers has created a highly interconnected decision environment where social proof is readily available and actively sought (Zhang et al., 2022; Zhao et al., 2022). Chinese consumers, aligned with their cultural scores, demonstrate greater adaptability and a stronger reliance on extensive social networks, such as online communities and influencer recommendations, for decision validation. In contrast, South Korea displays stronger elements of horizontal collectivism, where in-group harmony and equality are emphasized. Consequently, South Korean consumers exhibit stronger in-group orientation and higher uncertainty avoidance, preferring familiar information sources like friends and family, and favoring risk-averse strategies that rely on expert opinions and brand reputation (Jeong et al., 2019; Lee et al., 2022). This reflects Korea’s stronger reliance on close-knit social ties rather than broad, anonymous social proof. Therefore, the FOBO-to-ditto consumption path should be stronger for Chinese consumers, driven by broad social proof, and weaker for Koreans, tempered by cautious in-group norms. The following hypothesis is proposed:

**H7.**  *In the mediating pathway of decision uncertainty (a. obsolescence risk, b. choice overload, c. price fluctuation, d. time pressure) affecting ditto consumption via FOBO, cultural differences moderate the relationship between FOBO and ditto consumption.*

## 3. Research Methodology

### 3.1. Research Model

**Figure 1:**
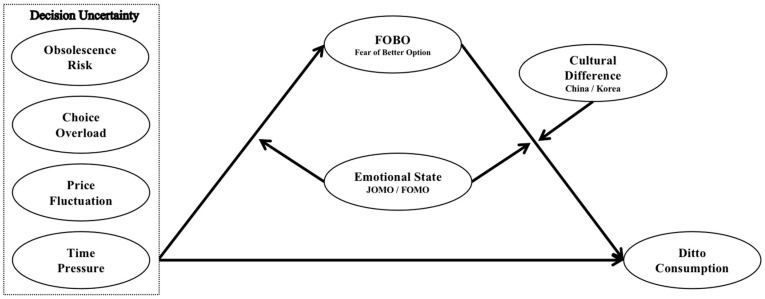
Research model. *Source: Authors’ own creation.*

This study employs a quantitative design using a structured questionnaire to examine how obsolescence risk, choice overload, price fluctuation, and time pressure influence FOBO and ditto consumption, and to assess the moderating effects of emotional state and cultural differences. A cross-sectional survey captured consumers’ decision tendencies, and SEM tested the proposed hypotheses and relationships among variables (Figure 1).

### 3.2. Procedure

The empirical analysis was conducted using SEM via AMOS 26.0 and SPSS 26.0. Given the rapid iteration, price volatility, and perceived quality uncertainty of new energy vehicles, their consumers often face heightened decision-making stress, making them a suitable population for examining FOBO and ditto consumption. Accordingly, the study focused on Chinese and Korean consumers who had purchased new energy vehicles within the past month, ensuring that responses were grounded in fresh consumption experiences. To capture authentic behavioral data, all surveys were administered through offline intercept sampling from June to July 2025. Trained research assistants approached new energy vehicle (NEV) owners in shopping mall parking lots in Beijing and Seoul and invited them to complete the questionnaire based on their latest purchase decision. These two capital cities were selected because they represent the most mature, policy-driven, and high-adoption NEV markets in their respective countries, thereby providing access to informed consumers who face the full spectrum of decision uncertainties. To encourage participation, respondents received a small gift as appreciation for their time.

### 3.3. Measures

The measurement of all variables in this study is based on validated scales from previous research, with adjustments for Chinese and Korean contexts. Obsolescence risk reflects the consumer’s concern that a product may soon become outdated or devalued due to rapid technological iterations or the launch of improved models. It is measured by consumers’ concern about new product releases shortly after purchase, hesitation to buy due to rapid technological advancement, belief that a chosen product may quickly become obsolete, and tendency to delay purchases until a new model is announced (Acikgoz et al., 2025; Guillard et al., 2023). Choice overload reflects consumers’ cognitive burden when making decisions amid excessive product options. It is measured by assessing consumers’ feelings of being overwhelmed by too many choices, delaying decisions due to choice abundance, experiencing mental fatigue from excessive comparisons, and reduced purchase satisfaction due to excessive alternatives (Hunter et al., 2024; Jacob et al., 2024). Price fluctuation reflects the impact of price volatility on consumer decisions. It is measured by consumers’ tendency to delay purchases in anticipation of price drops, difficulty making final decisions due to price uncertainty, post-purchase regret when encountering lower prices, and hesitation due to frequent price changes (Bai & Kim, 2024). Time pressure reflects the influence of perceived time constraints on consumer decision-making. It is measured by consumers’ tension when making decisions under limited time, decision delays due to time constraints, preference for quick selections over in-depth analysis, and anxiety caused by time pressure (Liu et al., 2022; Wu et al., 2025). FOBO refers to consumers’ hesitation or decision abandonment due to the belief in better alternatives. It is measured by consumers’ indecision caused by the belief in better alternatives, decision delays due to fear of suboptimal choices, anxiety from potential superior substitutes, and procrastination in decision-making due to choice difficulty (Flecha Ortiz et al., 2024; Park & Kim, 2025). Ditto consumption refers to the consumer behavior of relying on social validation to simplify decision-making. It is measured by assessing consumers’ tendency to mimic others’ choices under uncertainty, dependence on product reviews and ratings, increased decision confidence when choosing best-selling products, and reduced cognitive effort due to conformity (Kang et al., 2020; J. Wang et al., 2023). Emotional state includes JOMO, which reflects consumer satisfaction from avoiding impulsive or unnecessary purchases, and FOMO, which describes consumer anxiety over missing out on popular or trending products. To ensure the validity of JOMO and FOMO as opposing concepts, emotional state is measured by assessing consumer anxiety over missing out on trending information, mindset when browsing social media, feelings after friends adopt new trends, and satisfaction from following popular trends. Given that JOMO and FOMO are conceptually antithetical, an individual cannot simultaneously experience high levels of both. Higher scores indicate stronger FOMO, while lower scores indicate stronger JOMO (Chan et al., 2022; Eitan & Gazit, 2024). Cultural differences refer to variations in consumers’ psychological responses based on nationality. Cultural differences are measured using a nominal scale that classifies respondents into two groups, Chinese and Korean consumers. Survey items were translated into Chinese and Korean using a parallel translation method. A pre-test with 100 offline responses was conducted to refine the questionnaire. Ambiguities were revised based on the pre-test results, forming the final questionnaire.

### 3.4. Participants

A total of 750 surveys were collected, of which 682 were valid after removing 68 invalid responses, yielding a 90.93% effective rate. The sample showed a balanced gender distribution (52.3% female, 47.7% male) and a nearly even split between Chinese (51.3%) and Korean (48.7%) respondents. Full-time employees comprised 67.7% of the sample. Most participants (85.6%) held at least a bachelor’s degree, indicating a generally high education level. The majority (77%) were aged 20–49, representing young and middle-aged groups. Income levels were diverse, with 27.3% earning $800–$1200 per month and 21.8% exceeding $2000.

## 4. Results

### 4.1. Common Method Bias Test

To assess the potential threat of common method variance due to the cross-sectional, self-report design, this study conducted Harman’s one-factor test (Podsakoff et al., 2003). An unrotated exploratory factor analysis on all measurement items revealed that the first factor accounted for 25.736% of the total variance, which is below the recommended threshold of 50%. This result suggests that common method bias does not pose a major threat to the validity of the findings.

### 4.2. Measurement Model Analysis and Measurement Invariance Testing

The analysis confirmed the suitability of the data for factor analysis (KMO = 0.890; Bartlett’s test, *p* = 0.000). Six factors were extracted, with factor loadings ranging from 0.739 to 0.940, supporting the rational structure and strong convergent validity of the scale. In terms of reliability, Cronbach’s α (0.875–0.942) and CR values (0.864–0.957) exceeded the recommended threshold of 0.7, indicating high internal consistency. Regarding validity, AVE values ranged from 0.615 to 0.848, surpassing the 0.5 benchmark and confirming good convergent validity. Furthermore, the model demonstrated a good overall fit (χ^2^(237) = 765.454, GFI = 0.917, CFI = 0.958, TFI = 0.931, RMSEA = 0.057). In summary, the measurement scales met academic standards for reliability and validity, providing a solid basis for subsequent analysis.

To ensure the validity of subsequent cross-cultural comparisons, measurement invariance across the Chinese and Korean samples was tested using a series of multi-group CFA models. Configural invariance was supported, indicating consistent factor structures across groups. Constraining factor loadings resulted in negligible change in model fit (ΔCFI < 0.010), and further constraining item intercepts also met recommended thresholds (ΔCFI < 0.015). These results provide evidence of partial scalar invariance, supporting the appropriateness of cross-cultural path comparisons in later analyses (Table 1).

To assess discriminant validity, this study compared the square roots of the AVEs with the inter-construct correlations. The results indicate that the square roots of the AVEs (0.785–0.921) were greater than the corresponding correlation coefficients, and the confidence intervals of the correlations did not include 1.0. Although the correlation between FOBO and ditto consumption (r = 0.701) is relatively high, it remains below the square root of the AVE for each construct (0.784 and 0.785, respectively), satisfying the discriminant validity criterion. These results provide strong support for the discriminant validity of the constructs, consistent with the Fornell–Larcker criterion (Table 2).

### 4.3. Hypothesis Testing

#### 4.3.1. Direct Path Effect Analysis

The results show that obsolescence risk had no significant effect on FOBO or ditto consumption (*p* > 0.05); thus, H1a and H2a were not supported. Choice overload was significantly positively associated with FOBO (β = 0.397, *p* < 0.001) and ditto consumption (β = 0.272, *p* < 0.001), supporting H1b and H2b. Similarly, price fluctuation significantly predicted FOBO (β = 0.373, *p* < 0.001) and ditto consumption (β = 0.159, *p* < 0.001), confirming H1c and H2c. These results align with prior findings that choice overload (Jacob et al., 2024) and price fluctuations (Bai & Kim, 2024) heighten consumer uncertainty and contribute to decision avoidance behaviors. Interestingly, the effect of time pressure on FOBO was contrary to the hypothesized direction (β = −0.080, *p* = 0.005), rejecting H1d. However, time pressure had a significant positive effect on ditto consumption (β = 0.074, *p* = 0.004), supporting H2d, and echoing Begho and Liu (2024) that time-constrained consumers often rely on social proof to simplify decision-making. Notably, FOBO exerted the strongest effect on ditto consumption (β = 0.496, *p* < 0.001), supporting H3 and the view of Flecha Ortiz et al. (2024) that high fear is associated with consumers to seeking collective validation in decision-making (Table 3).

#### 4.3.2. Mediation Analysis

This study used Bootstrap Model 64 (5000 samples, 95% CI) to test mediation effects, examining the dual-path mechanism through which decision uncertainty influences ditto consumption. Following Preacher and Hayes (2004), mediation is significant when the 95% CI excludes zero. Given the cross-sectional design, this analysis tests a theoretically informed indirect association.

First, the analysis of the direct effect of obsolescence risk on ditto consumption was not significant (β = −0.012, *p* = 0.636, 95% CI [−0.063, 0.039]). Additionally, the indirect effects across all conditions of cultural differences and emotional state included zero (FOMO + China: β = 0.031, 95% CI [−0.030, 0.092]), indicating that H4a was not supported.

Second, the impact of choice overload demonstrated a typical partial mediation effect. The direct effect was significant (β = 0.272, 95% CI [0.215, 0.330]), and the mediated path through FOBO exhibited cultural differences. Specifically, the effect of FOMO-prone Chinese consumers on ditto consumption was the strongest (β = 0.276, 95% CI [0.215, 0.335]), significantly higher than that of JOMO-prone Korean consumers (β = 0.167, 95% CI [0.107, 0.241]). This result echoes the strong correlations among choice overload, FOBO, and ditto consumption (r = 0.568–0.622), confirming the anxiety-driven transmission mechanism of decision complexity.

Third, the effect of price fluctuation also demonstrated a typical partial mediation effect. The direct effect remained significant (β = 0.225, 95% CI [0.144, 0.306]), while the indirect effect peaked in the FOMO-prone Chinese consumers (β = 0.356, 95% CI [0.262, 0.453]), which was 42.4% higher than that of the JOMO-prone Korean consumers (β = 0.250, 95% CI [0.168, 0.348]). This cross-cultural difference may stem from the amplification of price sensitivity in collectivist cultures (Bai & Kim, 2024), forming a complete evidence chain alongside the moderate correlation between price fluctuation and FOBO (r = 0.460, Table 2).

Finally, time pressure produced the contradictory dual-path effect. The direct effect enhanced ditto consumption (β = 0.074, *p* = 0.004), but its indirect effect through FOBO was significantly negative in the JOMO group (China: β = −0.076, 95% CI [−0.139, −0.017]; Korea: β = −0.062, 95% CI [−0.116, −0.014]) while insignificant in the FOMO group. This result aligns with Gross’s (2015) emotion regulation theory, suggesting that JOMO individuals transform time pressure into decision-simplifying motivation, whereas FOMO individuals remain sensitive to social cues (Table 4). Cross-cultural comparisons further revealed that effect sizes were consistently larger among Chinese consumers than Korean consumers, possibly due to stronger conformity tendencies in collectivist cultures (Bai et al., 2024).

#### 4.3.3. Moderation Analysis

This study employed the Bootstrap method (Model 64, 5000 samples, 95% CI) to examine moderation effects, revealing how emotional state and cultural differences shape consumer behavior.

First, although the first-stage analysis indicated that emotional state did not significantly moderate the paths between decision uncertainty and FOBO (H5a–d: *p*s > 0.05), the second-stage analysis of the latter part of the mediation path (FOBO to DC) revealed significant selective moderation effects. Specifically, emotional state significantly enhanced the effects of obsolescence risk (β = 0.161, *p* = 0.007) and time pressure (β = 0.153, *p* = 0.010), while significantly weakening the effect of price fluctuation (β = −0.839, *p* = 0.002). This finding is broadly consistent with Gross’s (2015) emotion regulation theory, suggesting that individuals with FOMO exhibit a stronger “anxiety-to-ditto consumption” transmission under obsolescence risk and time pressure scenarios (FOMO β = 0.764, JOMO β = 0.603), whereas under price fluctuation conditions, they demonstrate greater resistance to price sensitivity.

Second, the study not only confirmed the stronger ditto consumption tendency among Chinese consumers (main effect β = 0.575, *p* = 0.037) but also further revealed that cultural differences generally weakened all three mediation paths (CO: β = −0.122; PF: β = −0.137; TP: β = −0.116, *p*s < 0.05). This indicates that while Chinese consumers exhibit a stronger overall tendency for ditto consumption, the effect of anxiety-driven paths is weaker. This paradox reflects the suppressive effect of social norms in collectivist cultures on emotion-driven behavior, aligning with interdependent-self theory. Particularly within the price fluctuation path, the simple slope in the Chinese sample (β = 0.698) was significantly higher than in the Korean sample, yet the moderation effect was negative, suggesting the possibility of nonlinear cultural influences.

Third, the study identified price fluctuation as a unique variable that was simultaneously dual-moderated by emotional state (β = −0.839, *p* = 0.002) and cultural differences (β = −0.137, *p* = 0.019). The FOMO + China combination exhibited the strongest effect (β = 0.698, *p* < 0.001). Interestingly, however, the overall ditto consumption level of the FOMO group was lower (β = −0.839, *p* = 0.002), forming what this study terms the Emotional State Paradox of Price Sensitivity as a descriptive observation. This finding enriches behavioral pricing theory, suggesting that cultural differences in price sensitivity may be mediated through differences in emotional state (Table 5).

**Figure 2:**
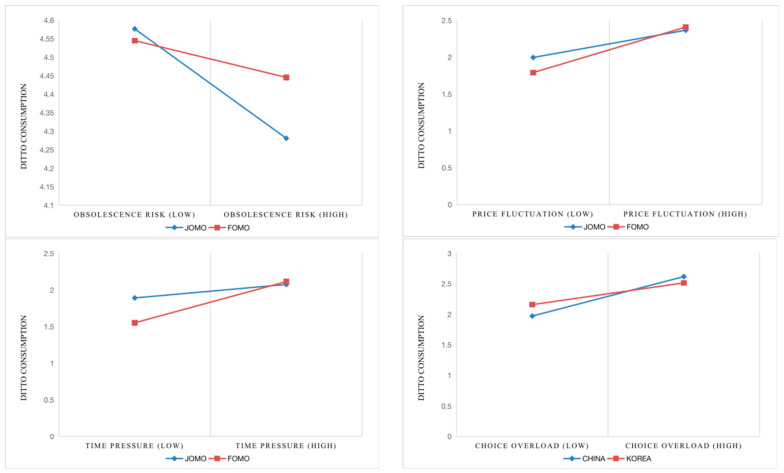

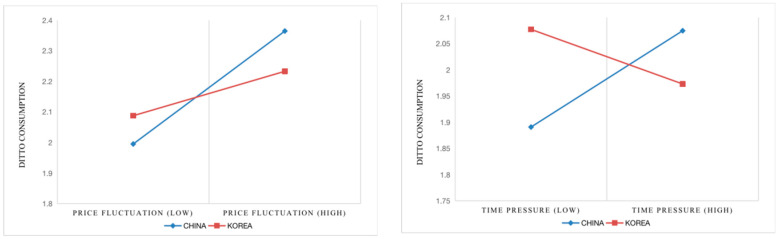
H6acb and H7bcd effect plot.

To enhance the interpretability of the significant moderation effects observed, visual representations of the results were created (Figure 2).

#### 4.3.4. Conditional Effects Analysis

Based on the above analysis, this study further explores the findings through a systematic conditional effects analysis, leading to the following conclusions. First, the study confirms that Chinese consumers exhibit stronger effect sizes across all psychological pathways (OR/CO/PF/TP) (mean Δβ = 0.125, *p*s < 0.001). Notably, in the time pressure pathway, the effect size for the Chinese FOMO group (β = 0.768, 95% CI [0.676, 0.859]) was 17.8% higher than that of the Korean counterpart (t = 3.21, *p* = 0.001). This finding aligns with Bai and Kim’s (2024) cultural dimensions theory, potentially reflecting the amplifying effect of collectivist cultures on time sensitivity. It is particularly noteworthy that the price fluctuation pathway exhibits the largest cultural difference (Δβ = 0.137), while emotional-state variation was smallest (FOMO-JOMO difference = 21.3%), implying that price-related decisions are shaped more by cultural values than transient emotions.

Second, FOMO significantly enhances cognitive-based pathways (OR/TP) more than situational-based pathways (CO/PF), with an average increase of 26.7% versus 19.5%, respectively (Fisher’s Z = 2.89, *p* = 0.004). This binary characteristic supports the dual-system theory, indicating that FOMO primarily amplifies decision-making processes based on individual cognitive evaluations. Under the FOMO + China combination, the time pressure pathway reaches its peak effect (β = 0.768), with a significant increase in explained variance (ΔR^2^ = 0.124, F = 9.87, *p* < 0.001), providing key empirical evidence for the cultural-emotional interaction theory (Table 6).

**Figure 3:**
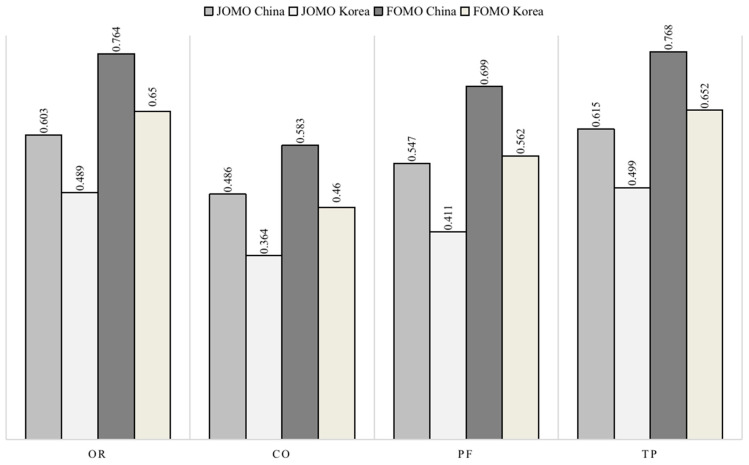
Conditional effects of moderating paths. Source: Authors’ own creation.

To clarify the distinct patterns among JOMO/FOMO and China/Korea consumers, the conditional effects on the dependent variable were visualized (Figure 3).

## 5. Discussion and Conclusions

### 5.1. Discussion

This study investigates how decision uncertainty factors influence FOBO and ditto consumption, with FOBO as a mediator and emotional state and culture as moderators. The findings confirm theoretical expectations while revealing new insights, enriching consumer decision-making theory. Results show that choice overload and price fluctuation significantly promote FOBO and ditto consumption, supporting choice paradox and information overload theories. However, obsolescence risk shows no significant direct effect, likely because rapid technological iteration in the NEV market has become a normative expectation that consumers already factor into their decisions (Guillard et al., 2023), suggesting it is not a primary driver in this context. Time pressure negatively affects FOBO but positively affects ditto consumption, a seemingly paradoxical pattern that can be reconciled by emotion regulation theory. Under time constraints, consumers suppress internal comparisons while relying on external social cues to simplify choices (Begho & Liu, 2024). This reflects consumers’ quick decisions and reliance on group opinions under time constraints. Mediation analysis reveals that FOBO partially mediates the effects of choice overload and price fluctuation on ditto consumption. Moderation analysis shows that FOMO amplifies, whereas JOMO mitigates FOBO’s effects. Moreover, significant differences exist between Chinese and Korean consumers in terms of effect sizes, highlighting the moderating role of cultural differences.

### 5.2. Theoretical Implications

First, this study provides initial evidence for FOBO as a potential mediating mechanism linking decision uncertainty to socially driven decision strategies. Prior research has separately examined antecedents of decision uncertainty and downstream coping behaviors (Alnes & Haugom, 2024; Bai & Kim, 2024; Begho & Liu, 2024). However, the literature has not articulated how uncertainty relates to socially grounded decision strategies. FOBO’s specific focus on the perceived existence of a superior alternative distinguishes it from anticipated regret or maximization tendency. This study positions FOBO as a psychological conduit through which decision uncertainty is associated with ditto consumption. By empirically supporting an “uncertainty–FOBO–ditto” chain across cultural contexts, this study offers a FOBO-centered perspective that helps explain why consumers may shift from individual evaluation to social validation. While FOBO can lead to multiple coping responses, this study focuses on ditto consumption as one prominent form of social validation. This mechanism enriches Social Identity Theory by suggesting that social conformity in high-load environments may arise not only from normative pressure but also from fear-driven cognitive overload.

Second, this study observes a dual-pathway effect of time pressure and suggests a fast-simplification heuristic as a tentative interpretation. Time pressure has historically been conceptualized as intensifying stress, accelerating cognitive processing, or impairing evaluation quality (Begho & Liu, 2024; Wu et al., 2025). The present findings challenge and refine this assumption by demonstrating a dual-pathway response: time pressure attenuates FOBO yet simultaneously strengthens ditto consumption. This divergence suggests that consumers under temporal constraint may employ a fast-simplification heuristic, reducing internal comparison processes while increasing reliance on external social cues. By identifying this novel psychological shortcut, the study offers insights relevant to dual-process theories and contributes to the literature by suggesting that time pressure does not uniformly escalate anxiety but instead may trigger selective deactivation of internal maximization motives and compensatory reliance on collective signals. This highlights a previously unrecognized mechanism in high-speed decision environments.

Third, this study suggests emotional state as an important boundary condition influencing FOBO transmission. Although prior work has linked FOMO and JOMO to digital behaviors, their role in moderating uncertainty-induced decision strategies has been largely unexplored (Park & Kim, 2025; Tan et al., 2025). This study demonstrates that emotional state selectively amplifies or suppresses the FOBO–ditto consumption pathway, FOMO magnifies the transmission of uncertainty-induced fear into imitation behavior, whereas JOMO buffers this transmission by promoting reflective disengagement. These findings extend emotion-regulation theory by illustrating that emotional predispositions act as meta-evaluative filters that modulate the motivational force of FOBO. The results also nuance the FOBO literature, showing that fear-driven decision tendencies are not uniform but contingent upon individuals’ emotional orientations toward social information. This suggests emotional state as a key boundary condition in fear-based decision models.

Finally, this study suggests a potential cultural norm suppression mechanism that may help explain intra-East Asian differences in uncertainty-driven decision patterns. Cross-cultural consumer research has often relied on macro-level distinctions while overlooking intra-East Asian subtleties (Deng & Gao, 2025; Truong, 2025). By comparing Chinese and Korean consumers, this study reveals a counterintuitive pattern: Chinese consumers display stronger overall tendencies toward ditto consumption, yet the FOBO-to-ditto pathway is weaker, suggesting that cultural norms of social harmony and risk-sharing may suppress the emotional transmission of FOBO. This study tentatively refers to this phenomenon as a potential cultural norm suppression mechanism, whereby collectivist contexts may promote imitation behavior while simultaneously dampening the psychological weight of individual anxiety. This contribution deepens cross-cultural consumer theory by illustrating that collectivism can simultaneously activate behavioral conformity and inhibit affective contagion, highlighting a more complex interplay between culture, emotion, and social decision strategies than previously theorized.

### 5.3. Managerial Implications

First, firms can reduce FOBO by structuring choice environments and stabilizing information. Since FOBO serves as the primary mechanism through which uncertainty undermines decision confidence, companies should prioritize choice-environment simplification and information stability. Reducing unnecessary assortment complexity, limiting rapid price fluctuations, and presenting clear attribute comparisons can substantially decrease consumers’ fear of missing out on superior alternatives. Decision aids such as recommended options, curated bundles, and transparent ranking cues can further mitigate FOBO and help consumers commit to choices more decisively. Platforms prone to overload, such as e-commerce sites and app marketplaces, may benefit from deploying guided decision flows that prevent cognitive overwhelm and reduce abandonment.

Second, firms can leverage time pressure as a behavioral accelerator while minimizing cognitive friction. The dual-pathway effect of time pressure suggests that moderate, well-framed time constraints can motivate faster decisions without inducing counterproductive anxiety. Managers should use time pressure strategically, emphasizing clear call-to-action cues and minimizing cognitively heavy comparisons. Limited-time promotions may accelerate purchase behavior, but only when accompanied by simplified choice architecture that directs attention toward socially validated options. The fast-simplification heuristic identified in this study indicates that time-sensitive campaigns should be paired with social proof elements such as real-time popularity indicators or peer usage statistics to guide consumers’ rapid evaluations.

Third, companies should tailor communication strategies to consumers’ emotional orientation (FOMO/JOMO). Emotional predispositions shape how consumers interpret and react to uncertainty. For FOMO-prone consumers, highlighting social engagement, community participation, or trending content can amplify decision certainty and increase conversion. Conversely, JOMO-prone consumers respond more positively to messages emphasizing control, minimalism, and stress-free decision environments. Personalizing marketing communication according to emotional profiles, detected through browsing behavior, engagement with social content, or platform-level emotional cues, enables firms to align decision contexts with consumers’ motivational orientations. This segmentation approach can reduce FOBO-driven hesitation and enhance the overall decision experience.

Finally, firms should adapt decision architectures to cultural norms and social expectations. The tentative interpretation of a potential cultural norm suppression mechanism suggests that managers operating in East Asian markets may need to consider both overt behavioral tendencies and underlying emotional responses. In markets such as China, where imitation behavior is relatively high but FOBO sensitivity is muted, firms can leverage community-based endorsements, KOL/KOC cues, and collective signals to guide consumer choices. In contrast, Korean consumers may exhibit stronger FOBO-driven sensitivity, requiring clearer reassurance mechanisms such as expert recommendations, risk-mitigating guarantees, and transparent product comparisons. Tailoring decision environments to these cultural tendencies strengthens persuasion effectiveness and reduces decision friction.

### 5.4. Limitations and Future Research

First, this study’s data were obtained solely from Chinese and Korean consumers. While the cross-cultural comparison revealed some cultural differences, the generalizability of the findings remains to be verified in other cultural contexts. Future research could expand to include more countries and regions to examine the applicability and variability of FOBO and its transmission mechanisms in different cultural and economic environments.

Second, the cross-sectional design of this study precludes strong causal inferences. Although our hypotheses were grounded in theory and the statistical mediation analyses support the proposed directional paths, alternative models cannot be ruled out. Future research should employ longitudinal designs or experimental manipulations to establish causality and to test whether ditto consumption actually alleviates FOBO or instead serves as a form of decision procrastination that deepens anxiety over time.

Third, while Harman’s one-factor test suggested that common method bias does not pose a major threat, common method variance cannot be completely eliminated in self-reported, cross-sectional surveys. Future studies could incorporate multi-source or time-lagged data to further mitigate this concern.

Fourth, the use of intercept sampling in shopping mall parking lots may introduce selection bias, as it primarily captures consumers who visit physical retail locations. Future research could complement this approach with online or randomized sampling strategies.

Fifth, this study measured emotional state on a single continuum from FOMO to JOMO, following prior literature. However, FOMO and JOMO may be independent constructs rather than opposite ends of a single dimension. Future research should measure FOMO and JOMO separately to examine their potentially distinct moderating effects on FOBO and ditto consumption.

Sixth, due to the absence of measures for general decision anxiety or indecision, this study was unable to conduct a competitive model test to assess FOBO’s incremental explanatory power over these related constructs. Although our conceptual and discriminant validity analyses support FOBO’s distinctiveness, future research should include parallel measures of general anxiety or indecision to formally test whether FOBO explains variance in ditto consumption beyond what these broader constructs capture.

Finally, although emotional state and cultural differences were introduced as moderating variables, other potential factors, such as FMCG or DCG product type and MBTI personality traits, were not considered. Future studies could incorporate these variables to develop a more comprehensive model of FOBO and ditto consumption and further explore their interaction mechanisms.

## Figures and Tables

**Table 1:** Confirmatory Factor Analysis Results.

Variables		Unstd.	S.E.	t-Value	FL	CR	AVE	Cronbach’s α
OR	OR1	1			0.894	0.954	0.839	0.937
	OR2	1.092	0.038	28.461 ***	0.898			
	OR3	1.200	0.035	33.994 ***	0.940			
	OR4	1.165	0.036	32.610 ***	0.930			
CO	CO1	1			0.839	0.892	0.673	0.903
	CO2	1.088	0.042	25.893 ***	0.841			
	CO3	1.094	0.045	24.316 ***	0.827			
	CO4	1.017	0.041	24.548 ***	0.773			
PF	PF1	1			0.830	0.891	0.671	0.875
	PF2	1.022	0.05	20.513 ***	0.811			
	PF3	1.078	0.05	21.586 ***	0.827			
	PF4	1.039	0.049	21.163 ***	0.808			
TP	TP1	1			0.924	0.957	0.848	0.942
	TP2	1.192	0.035	34.303 ***	0.915			
	TP3	1.087	0.030	36.101 ***	0.929			
	TP4	1.056	0.032	33.483 ***	0.915			
FB	FB1	1			0.798	0.864	0.615	0.893
	FB2	1.253	0.048	26.065 ***	0.774			
	FB3	1.215	0.044	27.734 ***	0.825			
	FB4	0.905	0.04	22.496 ***	0.739			
DC	DC1	1			0.755	0.865	0.616	0.896
	DC2	1.014	0.038	26.359 ***	0.804			
	DC3	1.057	0.044	23.974 ***	0.805			
	DC4	1.151	0.046	24.765 ***	0.775			

Notes: (1) χ^2^(237) = 765.454, GFI = 0.917, CFI = 0.958, TFI = 0.931, RMSEA = 0.057; (2) ***, *p* < 0.001; (3) OR (Obsolescence Risk), CO (Choice Overload), PF (Price Fluctuation), TP (Time Pressure), FB (FOBO), DC (Ditto Consumption).

**Table 2:** Discriminant Validity Results.

	AVE	DC	FB	TP	PF	CO	OR
DC	0.616	***0.785***					
FB	0.615	0.701	***0.784***				
TP	0.848	0.033	−0.094	***0.921***			
PF	0.671	0.465	0.460	0.065	***0.819***		
CO	0.673	0.622	0.568	−0.041	0.411	***0.820***	
OR	0.839	−0.009	0.004	0.136	0.021	0.010	***0.916***

Notes: (1) Diagonal values (bold/italics) are square root of AVE. (2) OR (Obsolescence Risk), CO (Choice Overload), PF (Price Fluctuation), TP (Time Pressure), FB (FOBO), DC (Ditto Consumption).

**Table 3:** Hypothesis Testing Results.

Hypothesis	Hypothesis Path	β	S.E.	C.R.	*p*	Results
H1a	OR→FB	0.006	0.030	0.195	0.845	Reject
H1b	CO→FB	0.397	0.034	11.794	***	Accept
H1c	PF→FB	0.373	0.047	7.947	***	Accept
H1d	TP→FB	−0.080	0.029	−2.797	0.005	Reject
H2a	OR→DC	−0.025	0.027	−0.941	0.347	Reject
H2b	CO→DC	0.272	0.034	8.021	***	Accept
H2c	PF→DC	0.159	0.044	3.643	***	Accept
H2d	TP→DC	0.074	0.026	2.882	0.004	Accept
H3	FB→DC	0.496	0.046	10.819	***	Accept

Notes: (1) χ^2^(243) = 879.267, GFI = 0.909, CFI = 0.949, TFI = 0.922, RMSEA = 0.062; (2) ***, *p* < 0.001. (3) OR (Obsolescence Risk), CO (Choice Overload), PF (Price Fluctuation), TP (Time Pressure), FB (FOBO), DC (Ditto Consumption).

**Table 4:** Mediating Effects Results.

Hypothesis		Effects	MV		β	S.E.	LLCI	ULCI	Results
H4a	OR→FB→DC	Direct			−0.012	0.026	−0.063	0.039	Reject
		Indirect	JOMO	China	−0.025	0.035	−0.093	0.044	
			JOMO	Korea	−0.020	0.028	−0.076	0.036	
			FOMO	China	0.031	0.031	−0.030	0.092	
			FOMO	Korea	0.026	0.027	−0.026	0.078	
H4b	CO→FB→DC	Direct			0.272	0.029	0.215	0.330	Partial mediation
		Indirect	JOMO	China	0.223	0.036	0.157	0.297	
			JOMO	Korea	0.167	0.034	0.107	0.241	
			FOMO	China	0.276	0.031	0.215	0.335	
			FOMO	Korea	0.218	0.033	0.155	0.284	
H4c	PF→FB→DC	Direct			0.225	0.041	0.144	0.306	Partial mediation
		Indirect	JOMO	China	0.333	0.048	0.247	0.434	
			JOMO	Korea	0.250	0.045	0.168	0.348	
			FOMO	China	0.356	0.048	0.262	0.453	
			FOMO	Korea	0.286	0.048	0.198	0.386	
H4d	TP→FB→DC	Direct			0.074	0.025	0.025	0.124	Partial mediation
		Indirect	JOMO	China	−0.076	0.031	−0.139	−0.017	
			JOMO	Korea	−0.062	0.026	−0.116	−0.014	
			FOMO	China	−0.023	0.034	−0.089	0.042	Reject
			FOMO	Korea	−0.019	0.028	−0.074	0.038	

Note: OR (Obsolescence Risk), CO (Choice Overload), PF (Price Fluctuation), TP (Time Pressure), FB (FOBO), DC (Ditto Consumption).

**Table 5:** Moderation Effects Results.

Hypothesis		DV	β	S.E.	t-Value	*p*	LLCI	ULCI	Results
H5a	OR*ES	FOBO	0.081	0.068	1.182	0.238	−0.053	0.215	Reject
H5b	CO*ES	FOBO	0.015	0.061	0.241	0.809	−0.105	0.135	Reject
H5c	PF*ES	FOBO	−0.100	0.093	−1.082	0.280	−0.282	0.082	Reject
H5d	TP*ES	FOBO	0.094	0.067	1.396	0.163	−0.038	0.226	Reject
H6a	OR(FB*ES)	DC	0.161	0.059	2.729	0.007	0.045	0.277	Accept
H6b	CO(FB*ES)	DC	0.096	0.056	1.718	0.086	−0.014	0.206	Reject
H6c	PF(FB*ES)	DC	−0.839	0.269	−3.113	0.002	−1.368	−0.310	Accept
H6d	TP(FB*ES)	DC	0.153	0.059	2.601	0.010	0.037	0.268	Accept
H7a	OR(FB*CD)	DC	−0.114	0.059	−1.929	0.054	−0.230	0.002	Reject
H7b	CO(FB*CD)	DC	−0.122	0.056	−2.201	0.028	−0.232	−0.013	Accept
H7c	PF(FB*CD)	DC	−0.137	0.058	−2.359	0.019	−0.251	−0.023	Accept
H7d	TP(FB*CD)	DC	−0.116	0.059	−1.977	0.048	−0.231	−0.001	Accept

Notes: OR (Obsolescence Risk), CO (Choice Overload), PF (Price Fluctuation), TP (Time Pressure), FB (FOBO), DC (Ditto Consumption), ES (Emotional State), CD (Cultural Differences). The “*” indicates the interaction term between two variables.

**Table 6:** Conditional Effects Analysis Results.

DV	IV	Moderating Variable		Effects	S.E.	t-Value	*p*	LLCI	ULCI
DC	OR	JOMO	China	0.603	0.054	11.253	0.000	0.498	0.708
		JOMO	Korea	0.489	0.048	10.179	0.000	0.395	0.583
		FOMO	China	0.764	0.047	16.297	0.000	0.672	0.856
		FOMO	Korea	0.650	0.055	11.904	0.000	0.543	0.757
	CO	JOMO	China	0.486	0.052	9.367	0.000	0.384	0.588
		JOMO	Korea	0.364	0.047	7.720	0.000	0.271	0.457
		FOMO	China	0.583	0.048	12.076	0.000	0.488	0.677
		FOMO	Korea	0.460	0.055	8.338	0.000	0.352	0.569
	PF	JOMO	China	0.547	0.053	10.253	0.000	0.443	0.652
		JOMO	Korea	0.411	0.049	8.358	0.000	0.314	0.507
		FOMO	China	0.699	0.047	14.724	0.000	0.605	0.792
		FOMO	Korea	0.562	0.056	10.083	0.000	0.452	0.671
	TP	JOMO	China	0.615	0.053	11.538	0.000	0.510	0.720
		JOMO	Korea	0.499	0.048	10.426	0.000	0.405	0.593
		FOMO	China	0.768	0.047	16.468	0.000	0.676	0.859
		FOMO	Korea	0.652	0.054	12.018	0.000	0.545	0.758

Note: OR (Obsolescence Risk), CO (Choice Overload), PF (Price Fluctuation), TP (Time Pressure).

## Data Availability

The data presented in this study are available on request from the corresponding author.
